# Primary hyperparathyroidism in pregnancy: experience of a tertiary centre

**DOI:** 10.1007/s00595-022-02583-8

**Published:** 2022-09-15

**Authors:** Muhammad Fahad Arshad, Maulee Hiromi Arambewela, William M. Bennet, Monique Sterrenburg, Saba P. Balasubramanian

**Affiliations:** 1 Oncology and Metabolism, University of Sheffield Medical School, Sheffield, S10 2RX UK; 2 Sheffield Teaching Hospitals, Royal Hallamshire Hospital, Sheffield, S10 2JF UK; 3 University of Sri Jayewardenepura, Nugegoda, Sri Lanka

**Keywords:** Primary hyperparathyroidism, Pregnancy, Surgery, Hypercalcaemia

## Abstract

**Background and purpose:**

The management of primary hyperparathyroidism (PHPT) during pregnancy is challenging and there is no clear consensus on whether it increases the risk of complications in pregnancy. We conducted this study to review the maternal and fetal outcomes of pregnant women treated for PHPT in a single centre.

**Methods:**

Data on relevant clinical parameters, demographics, management strategies, maternal and fetal outcomes were collected from the medical records of pregnant patients with PHPT diagnosed between 2012 and 2019.

**Results:**

Of 15 pregnant women with PHPT, 6 were managed medically and 9 underwent surgery. The median age at their index pregnancy was 28 years [range 19–42]. The median highest adjusted calcium level in the medical group was 2.90 [range 2.61–3.25] mmol/L vs. 3.11 [2.78–4.95] mmol/L in the surgical group. There was one miscarriage and the stillbirth of twins in the medical group, but no such outcomes in the surgical group. The median gestational ages were 39 + 3 weeks [range 24 + 2–41 + 2 weeks] and 39 + 4 weeks [range 37 + 1–39 + 5 weeks] in the medical and surgical groups, respectively. No birth was complicated by neonatal tetany or convulsions.

**Conclusion:**

More complications developed in the pregnant PHPT patients who were managed medically than in those who underwent surgery. Surgery performed during the second trimester resulted in good outcomes. Multi-centre prospective studies are required to ascertain the risk of various complications in women with PHPT during pregnancy.

## Introduction

Primary hyperparathyroidism (PHPT) is a relatively common endocrine disorder with an overall prevalence estimated at around 0.86% [1]. It is more common in women than in men, with a reported incidence of 66 per 100,000 patients per year vs. 25 per 100,000 patients per year, respectively [2]. It is asymptomatic in many patients and may remain undiagnosed for a long time. The incidence of PHPT among women of childbearing age is low, at around 8/100,000 patients per year, which rises to 188/100,000 patients per year in women over 40 years old [3]. One large study estimated that PHPT occurs in 1 in every 2000 (0.05%) women of reproductive age [4].

The management of PHPT during pregnancy is challenging for two important reasons. One is the consequence of high calcium on maternal and fetal outcomes, although its reported effects are conflicting. Some authors have reported maternal hypertension, pre-eclampsia, miscarriages, and pre-term labour being associated with hypercalcaemia [5–7], whereas others have not [4, 8]. Similarly, poor fetal outcomes, including stillbirth and intrauterine growth retardation, have been reported by some, [6, 9] but not by others [8]. The second reason is the potential risks of medical and surgical management of PHPT during pregnancy. Management decisions are complicated by the lack of evidence and, therefore, clarity on the best timing for medical or surgical intervention in pregnancy. This decision is made on a case-by-case basis; often based on local experience and availability of expertise; ideally by a multidisciplinary team (MDT), as suggested by the National Institute for Health and Care Excellence (NICE) in the UK [10]. Guidelines from the fourth international workshop support a surgical approach for patients under 50 years  of age, which will likely include all pregnant patients [11]. However, the guidelines do not discuss pregnancy or, more specifically, the timing of surgery in relation to pregnancy.

The usual practice in this centre is to discuss all pregnant PHPT patients in MDT meetings, basing recommendations on the degree of hypercalcaemia, the stage of pregnancy, and the presence of other co-morbidities. All patients are counselled on the potential effects of untreated PHPT on the mother and fetus and the relative risks and benefits of surgery vs. medical treatment. Generally, patients with mild hypercalcemia are managed conservatively during pregnancy, whereas those with severe hypercalcemia are encouraged to undergo surgery, ideally in the second trimester.

The aim of this study was to review the maternal and fetal outcomes of pregnant women treated for PHPT at a single tertiary care centre and compare our findings with those of similar studies published in the literature.

## Methods

All pregnant patients treated for PHPT at the Sheffield teaching hospitals in the UK between 2011 and 2019 were identified through clinicians’ logs and the operating database. Electronic and paper records were reviewed to collect data on the demographics, clinical features, biochemistry, management, and outcomes of PHPT during pregnancy. We analysed maternal outcomes, including hypertension or pre-eclampsia, gestational age at delivery, mode of delivery, and blood loss at labour; and fetal outcomes, including stillbirth or miscarriage, birth weight, and tetany or convulsions in the neonatal period. The data were collected and analysed using Microsoft Excel. All data were stored confidentially on hospital computers. Since this was a case series, a formal ethics application was not deemed necessary.

## Results

We identified 15 pregnant patients with PHPT: 10 with sporadic PHPT and 4 with multiple endocrine neoplasia type 1 (MEN1) syndrome, diagnosed by genetic testing prior to pregnancy. One of these patients had histologically diagnosed parathyroid cancer. The median age at their index pregnancy was 28 years (range 19–42 years). Table 1 summarizes the baseline characteristics of the 15 patients.

**Table 1:** Baseline characteristics of the pregnant women with primary hyperparathyroidism managed medically or surgically

	Medically managed (*n* = 6)	Surgically managed (*n* = 9)
Diagnosis	Sporadic = 4 MEN1 = 2	Sporadic = 6 MEN1 = 2 Parathyroid cancer = 1
Median age in years (range)	28 (19–42)	28 (19–37)
Ethnicity	Caucasian = 5, Black = 1	Caucasian = 8, Egyptian = 1
Parity	Primipara = 2 Multipara = 4	Primipara = 7 Multipara = 2
Smoking status	Smokers = 2, Non-smokers = 3, Unknown = 1	Smokers = 2, Non-smokers = 7
Median BMI in kg/m2 (range)	27 (22–32)	25 (20–31)
Number of babies	Single = 5, twin = 1	Single = 9
Median adjusted calcium (range) in mmol/L Normal range 2.2–2.6 [8.8–10.4 mg/dL]	2.90 (2.61–3.04) [11.6 (10.4–12.2)]	3.11 (2.78–4.95) [12.4 (11.1–19.8)]
Median PTH (range) in pmol/L Normal range 1.6–6-9 [15–65 ng/L or pg/mL]	12.0 (5.3–19.2) [113.2 (50.0–181.1)]	12.9 (9.2–82.8) [121.7 (86.8–780.8)]

*MEN* multiple endocrine neoplasia, *BMI* body mass index, *PTH* parathyroid hormone

Six patients were managed conservatively and 9 underwent parathyroid surgery during pregnancy. These surgeries were performed during the 2nd trimester in all except one patient whose PHPT was diagnosed at 30 weeks and who underwent surgery for severe hypercalcaemia at 31 weeks. Ultrasound of the neck was the sole modality for preoperative localisation in all of the patients treated surgically, except for one, who also had parathyroid four-dimensional computed tomography (4DCT) with abdominal shielding because of her complicated history. Bilateral neck exploration was performed in seven of the nine patients who were managed surgically, and unilateral neck exploration was performed in two. Supplementary Table 1 gives details of the preoperative localisation and intraoperative findings for these patients.

Of the six patients who were managed conservatively during pregnancy, four either refused surgery or missed appointments to discuss surgical treatment. The remaining two had PHPT diagnosed late in their pregnancy, and a decision was made to postpone surgery until after delivery. Five patients in this group underwent surgery after pregnancy, and one is awaiting surgery. These five patients underwent ultrasound and sestamibi scans before surgery. After informed consent and counselling regarding the uncertainty of the potential impact of cinacalcet on the fetus, cinacalcet was prescribed during pregnancy to one patient in each group. The serum calcium and PTH levels in the medically treated group were lower those in the surgical group. The patient with parathyroid cancer had the highest levels of calcium and PTH.

There was one miscarriage in the medical group but none in the surgical group. One twin pregnancy of a woman in the medical group resulted in stillbirth of both fetuses, although the intrauterine growth and development of these fetuses was severely impaired and the pregnancies were not thought to be viable, independent of the hypercalcaemia. None of the neonates of the mothers from either group had documented tetany or convulsions and their birth weights were comparable. Table 2 summarizes the maternal and fetal outcomes of the two groups. Among the women who underwent surgery during pregnancy, one had mild persistent disease (adjusted calcium 2.5–2.7 mmol/L), and one suffered long-term hypoparathyroidism requiring calcium and activated vitamin D supplementation. Both these patients are being monitored. There was no evidence of injury to the recurrent laryngeal nerve in any of the patients treated surgically; however, postoperative laryngoscopy was not performed routinely, in accordance with the findings of a recent study from the unit [12].

**Table 2:** Maternal and fetal outcomes in the medical and surgical groups of pregnant patients with primary hyperparathyroidism

	Medical (*n* = 6)	Surgical (*n* = 9)
Miscarriages	1	0
Hypertension/pre-eclampsia	2	2
Median gestational age at delivery in weeks (range)	39 + 3 (24 + 2–41 + 2) (*n* = 5 excluding miscarriage)	39 + 4 (37 + 1–39 + 5)
Induction of labour	1 (*n* = 5 excluding miscarriage)	4
Mode of delivery CS: C-section, EM: emergency, EL: elective, NVD: normal vaginal delivery	1 EM CS 4 NVD (*n* = 5 excluding miscarriage)	6 NVD 1 EL CS 1 EM CS 1 Instrumental (Ventouse)
Median blood loss at delivery in ml (range)	350 (150–800) (*n* = 5 excluding miscarriage)	400 (200–800) (2 unknown)
Medications required in peri-partum period	Steroids = 1 Antihypertensive drugs = 1	Steroids = 1 Antihypertensive drugs = 1
Stillbirth	2 (twin pregnancy)	0
Median birth weight in grams (Range)	3260 (260–4465)	3422 (2680–3940)
Neonatal tetany or convulsions	0	0

*CS* C-section, *EM* emergency, *EL* elective, *NVD* normal vaginal delivery

## Discussion

This is a report on the management of patients with PHPT during pregnancy based on the experience of a single centre. Patients with mild biochemical disease were managed conservatively, whereas those with significant hypercalcaemia underwent surgery in the second or early third trimester. Although limited by small numbers, our study shows comparable maternal and fetal outcomes between the two groups. However, there was one miscarriage and the stillbirth of twins in the medical group, although it is difficult to ascertain if hypercalcaemia resulted in or contributed to this.

Table 3 lists other published studies on the management and outcomes of cohorts of hyperparathyroid pregnant women. One large study by Norman et al. [7], which included 32 women and 72 pregnancies, raised concerns about fetal loss, reporting that 30 (48%) of 62 pregnancies managed medically ended in miscarriage. Such findings were not reported by other studies. Abood et al. [8] found no evidence of increased risk of low birth weight or abortion of PHPT compared with ‘no PHPT’ in a retrospective cohort study from a Danish registry of pregnant women. Another study including 74 women and 124 pregnancies [4], also found no risk of increased pregnancy-related complications, including miscarriage in patients with PHPT. There were two miscarriages in a study of 22 patients (28 pregnancies) [5] but, as in our study, these were not attributable to PHPT. These two latter studies, however, compared outcomes in pregnant women with PHPT with those without PHPT, without stratifying outcomes by medical management.

**Table 3:** Key studies describing the outcomes of medical and surgical treatment of primary hyperparathyroidism in pregnancy

Study	Number of patients in the study	Main outcomes reported
Kelly et al. [1]	12 patients Medical = 4, Surgical = 8	•All neonates born to mothers treated medically had hypocalcaemia and tetany •No complications in the surgical group
Norman et al. [7]	32 patients (77 pregnancies) Medical = 62, Surgical = 15	•3.5-fold increase in fetal loss among medically treated patients •No complications in the surgical group
McMullen et al. [11]	7 patients Medical = 3, Surgical = 4	•1 miscarriage and 2 pre-term deliveries in the medical group •No complications in the surgical group
Rigg et al. [5]	22 patients (28 pregnancies) Medical = 22, Surgical = 6	•Increased risk of preeclampsia and pre-term delivery in the medical group. Two miscarriages and one mild asymptomatic neonatal hypocalcaemia in the medical group. No complications in the surgical group

*FHH* familial hypocalciuric hypercalcemia

Maternal hypertension and pre-eclampsia have been associated with PHPT in several studies [5, 13]. Although these associations were not identified in our study or in a study by Hirsch et al. [4], links between hypertension, cardiovascular disease, and preeclampsia with PHPT in pregnancy are well documented [14–16]. Neonatal hypocalcemia with tetany has been reported historically [1], but fortunately, this is reported less frequently in recent studies [5, 13].

Consistent with findings in this report, other studies in the literature report good pregnancy-related outcomes for patients treated with parathyroid surgery during pregnancy [1, 5, 7, 17]. Surgical case series without control groups of patients managed conservatively during pregnancy have also confirmed this finding [18, 19]. Decisions regarding medical or surgical treatment were guided by MDT discussions in these two studies, as in this cohort, but we note that some patients in these studies underwent surgery despite having only mild hypercalcaemia (< 2.85 mmol/L) [16]. Based on observational data, it appears that the success rate of parathyroid surgery and other surgery-related outcomes are lower in the pregnancy cohort than in the general population [20, 21]. There may be several reasons for this including the limitations of preoperative imaging in pregnant patients and the higher likelihood of multi-gland disease in this population.

Cinacalcet was given to two patients in our cohort after careful counselling. The first had undergone two unsuccessful parathyroid operations elsewhere prior to her pregnancy and was referred to our institute for further management. A trial of cinacalcet was given for severe hypercalcaemia, pending investigations and decisions on further surgery. The second patient was given cinacalcet to reduce the severity of her hypercalcaemia after she declined the option of surgery. The use of cinacalcet in this situation is reported to be safe [22, 23]; however, the recent UK NICE guidelines do not recommend its use in pregnancy [10].

The main limitations of this study are the small number of patients and its retrospective design. Since serum calcium testing is not done routinely during pregnancy, it is likely that some hypercalcaemic pregnant patients remain undiagnosed. While we assessed all patients and performed surgery in one hospital, subsequent conservative management was undertaken in different hospitals resulting in some missing data.

## Conclusions

1. The management of hypercalcemia in pregnancy remains challenging in the absence of defined guidelines and the limitations of imaging for preoperative localisation.
2. More complications occurred in medically treated patients; however, the evidence for this association is limited. Multi-centre prospective studies are required to confirm or refute these findings.
3. Surgery during pregnancy is generally performed during the second trimester with good outcomes; therefore, a surgical treatment option should be considered for all pregnant patients with significant hypercalcaemia from PHPT.

## Future research questions

- What is the incidence of hypercalcaemia in pregnancy and what is the proportion of cases with primary hyperparathyroidism as the underlying cause in pregnant women with hypercalcaemia?
- What are the effects of various degrees of hypercalcaemia on maternal and fetal outcomes?
- What are the long-term effects of hypercalcaemia; for example, on bone health, in children born to mothers who had hypercalcaemia during pregnancy?

## Data Availability

All data generated or analysed during this study are included in this published article.

## Abbreviations

PHPT: Primary hyperparathyroidism
MDT: Multidisciplinary team
NICE: National Institute for Health and Care Excellence
MEN1: Multiple endocrine neoplasia type 1
